# Direct ECL Detection of Fentanyl Drug with Bare Screen-Printed Electrodes

**DOI:** 10.3390/bios15100697

**Published:** 2025-10-15

**Authors:** David Ibáñez, María Begoña González-García, David Hernández-Santos, Pablo Fanjul-Bolado

**Affiliations:** Metrohm DropSens S.L.U., Parque Tecnológico de Asturias, C/Faya 28, 33428 Llanera, Spain; begona.gonzalez@metrohm.com (M.B.G.-G.); david.hernandez@metrohm.com (D.H.-S.)

**Keywords:** electrochemiluminescence (ECL), fentanyl, tris(2,2′-bipyridyl)ruthenium(II) (Ru(bpy)_3_^2+^), screen-printed electrodes

## Abstract

Electrogenerated chemiluminescence (ECL) is a powerful analytical technique that combines the best features of both electrochemical and photoluminescence methods. In this work, we present a direct ECL-based method for the detection of fentanyl using unmodified screen-printed electrodes. The analysed system consists of tris(2,2′-bipyridyl)ruthenium(II) (Ru(bpy)_3_^2+^) as the luminophore and fentanyl as the co-reactant. A comprehensive optimization of the experimental parameters, such as buffer pH, luminophore concentration and working electrode material, was performed in order to maximize the ECL response. The optimal conditions are identified as PBS buffer pH 6, 2.5 × 10^−3^ M Ru(bpy)_3_^2+^ and bare gold screen-printed electrodes. Under these conditions, the system exhibited a strong and reproducible ECL signal, with a linear response to fentanyl concentration from 1 × 10^−7^ to 1 × 10^−5^ M and a limit of detection of 6.7 × 10^−8^ M. Notably, the proposed method does not require electrode surface modification, sample pretreatment or complex instrumentation, offering a rapid, sensitive, and cost-effective alternative for fentanyl detection. Furthermore, the storage of bare SPEs at room temperature in a dry place ensures their stability over months or even years, overcoming the limitations offered by ECL systems based on modifications of the working electrode with different nanomaterials. These findings highlight the potential of the proposed ECL approach as a robust and sensitive tool for the detection of synthetic opioids. Its simplicity, portability, and analytical performance make it particularly attractive for forensic and clinical applications where rapid and accurate opioid screening is essential.

## 1. Introduction

Electrochemiluminescence (ECL), also known as electrogenerated chemiluminescence, is a powerful analytical technique based on the emission of light when an electrochemical reaction produces the excitation of a luminophore (molecule which emits light) in the presence of a co-reactant [1]. Typically, ECL experiments follows the sequence of: (1) application of electrochemical conditions (potential/current) to the working electrode, (2) generation of reactive intermediates during the oxidation/reduction reactions of the luminophore or the co-reactant, (3) formation of an excited state close to the electrode surface upon interaction of intermediates and (4) emission of light when the excited state returns to the ground state. This mechanism provides the high sensitivity and selectivity required for analytical applications. Among its most notable strengths are its exceptionally low background signal and its high sensitivity, which significantly enables the detection of analytes at trace levels [2,3]. This technique also offers precise position and time control of the luminescent signal since the emission of light is produced on the working electrode surface due to the application of specific potentials/currents [4,5]. Moreover, its versatility is further demonstrated by the wide range of compatible luminophores (ruthenium complexes, luminol, quantum dots, etc.) [6] and co-reactants (H_2_O_2_, NADH, tris(2-pyridylmethyl)amine, triethylamine, dibutylaminoethanol, etc.) allowing a huge variety of applications in biosensing and clinical diagnostics [7,8,9], pharmaceutical and drug detection [10,11], food safety and environmental monitoring [12,13,14], material science [14,15,16] and forensic chemistry [17,18,19]. The efficiency of ECL emission is highly dependent on the kinetics of intermediate formation and the proximity of the excited species to the electrode surface. In this way, ruthenium-based complexes such as tris(2,2′-bipyridyl)ruthenium(II) exhibit strong and stable luminescence, making them ideal for sensing applications. Additionally, ECL systems benefit from simple and cost-effective instrumentation, as they require only an electrochemical cell and a detector. ECL is well-suited for miniaturization and integration into microfluidic and lab-on-a-chip platforms, supporting the development of portable and point-of-care diagnostic devices [20].

Fentanyl is a synthetic opioid known to be approximately 50 times stronger than heroin and 100 times than morphine [21,22]. Its powerful analgesic properties make pharmaceutical-grade fentanyl suitable for managing severe pain, such as that post-surgery or in advanced-stage cancers. However, patients with prescribed fentanyl require careful monitoring as it plays a significant role in overdose incidents. Illegally manufactured fentanyl can be found in various forms, including powders and liquids. It is frequently combined with other illicit substances, such as heroin, cocaine, or methamphetamine, and pressed into pills that resemble prescription opioids or other drugs [21]. Fentanyl test strips are widely used as a low-cost harm reduction tool to help prevent opioid overdoses. Although these strips can detect the presence of fentanyl in illicit substances, this method typically requires between three to five minutes to provide the results, with this delay being critical in case of overdoses. Moreover, drug safety is not guaranteed after obtaining negative results since many fentanyl analogues or more potent synthetic opioids may go undetected. Additionally, these strips provide only qualitative information, indicating the presence or absence of fentanyl without quantifying its concentration. Given these limitations, the need for the development of rapid, sensitive, and selective detection methods for fentanyl and its analogues is clear.

Although the electrochemical detection of fentanyl has been previously investigated [23,24,25,26,27,28,29,30,31,32,33,34,35], not only the electrochemical sensors but also ECL approaches [36] have been limited to electrodes specifically modified for this purpose. In this work, we proposed an ECL method for the detection of fentanyl using bare commercial electrodes. The optimization of the experimental conditions (pH, luminophore concentration, working electrode and electrochemical conditions) was performed to achieve the best results. The proposed protocol does not require the modification of the working electrode surface, the pretreatment of the sample nor long measurements. In this way, it offers an interesting alternative to the traditional methods, combining simplicity with high analytical performance.

## 2. Materials and Methods

### 2.1. Reagents

Fentanyl CII (USP Reference standard), tris(2,2′-bipyridyl)dichlororuthenium(II) hexahydrate (Rubpy_3_Cl_2_, Sigma-Aldrich, St. Louis, MI, USA), acetaminophen (Sigma-Aldrich, USA), L-ascorbic acid (Sigma-Aldrich, USA), caffeine (Sigma-Aldrich, USA), D-(+)-glucose (Sigma-Aldrich, USA), urea (Sigma-Aldrich, USA), sodium chloride (Sigma-Aldrich, USA), potassium chloride (Sigma-Aldrich, USA), sodium phosphate dibasic (Sigma-Aldrich, USA) and potassium phosphate monobasic (Sigma-Aldrich, USA) were used as received. All chemicals were analytical grade. Aqueous solutions were prepared using ultrapure water (Direct-QTM 5 system, Millipore, Hayward, CA, USA).

### 2.2. Instrumentation

Carbon, single-walled carbon nanotubes (SWCNTs) and platinum and gold screen-printed electrodes (110, 110SWCNT, 550BT, 220AT SPEs, respectively, Metrohm DropSens, Llanera, Spain) were evaluated as ECL platforms. The electrodic system consists of a flat ceramic card with a circular carbon/SWCNT/platinum/gold working electrode (4 mm diameter), a carbon (110 and 110WCNT), platinum (550BT) or gold (220AT) auxiliary electrode and a silver pseudo-reference electrode. The morphology of these SPEs was characterized using a scanning electron microscope (JEOL-6610LV, JEOL, Tokyo, Japan). Since the SPEs are used without any surface modification, stability issues are minimized. Unmodified electrodes were stored at room temperature in a dry environment, and consistent electrochemical signals were obtained even after one year, confirming their long-term stability.

**Figure 1:**
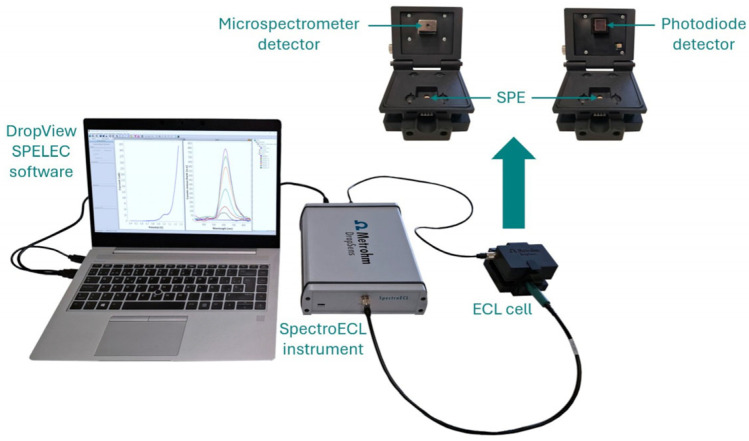
Experimental setup consists of the ECL instrument in combination with the microspectrometer or photodiode cell. The detector is as close as possible to the working electrode surface for the efficient collection of the generated emission.

ECL measurements were performed at room temperature using SpectroECL instrument (SPECTROECL, Metrohm DropSens, Spain) with a microspectrometer cell as detector. As can be seen in Figure 1, the easy experimental setup facilitates the performance of ECL experiments. The combination of the instrument and this cell allows the simultaneous acquisition of the electrochemical response and the emission spectra in the visible range (340–850 nm) as well as ensures the synchronization of both signals. Additionally, the instrument was also used with a photodiode detector cell (ECLPHOTODIODCELL, Metrohm DropSens, Spain). This device provides high sensitivity towards very low-level light signals, which is particularly important in the detection of low concentrations of one analyte. Data acquisition as well as data treatment were done with DropView SPELEC 3.69.3 software.

### 2.3. Methods

All ECL experiments were carried out at room temperature. A systematic evaluation of experimental parameters was performed to optimize the ECL response for fentanyl detection. Among the tested conditions, linear sweep voltammetry from +0.40 V to +1.30 V, scanning the potential at 0.05 V·s^−1^ in 0.1 M PBS solution (pH 6), provides the most reproducible and intense luminescent signals. This potential range was selected based on the redox behaviour of the luminophore and co-reactant system, ensuring the generation of reactive intermediates necessary for efficient excited-state formation. Emission spectra were recorded using the microspectrometer cell with an integration time of 3 s. These conditions enable the collection of wavelength-resolved data, facilitating the identification of the emission bands during the electrochemical reaction. ECL signals recorded with the photodiode cell were obtained applying the amplification factors ×10 and ×100. These amplification settings were chosen to acquire both low- and high-intensity signals under optimized parameters, without saturating the detector.

## 3. Results

### 3.1. Characterization of ECL System

**Figure 2:**
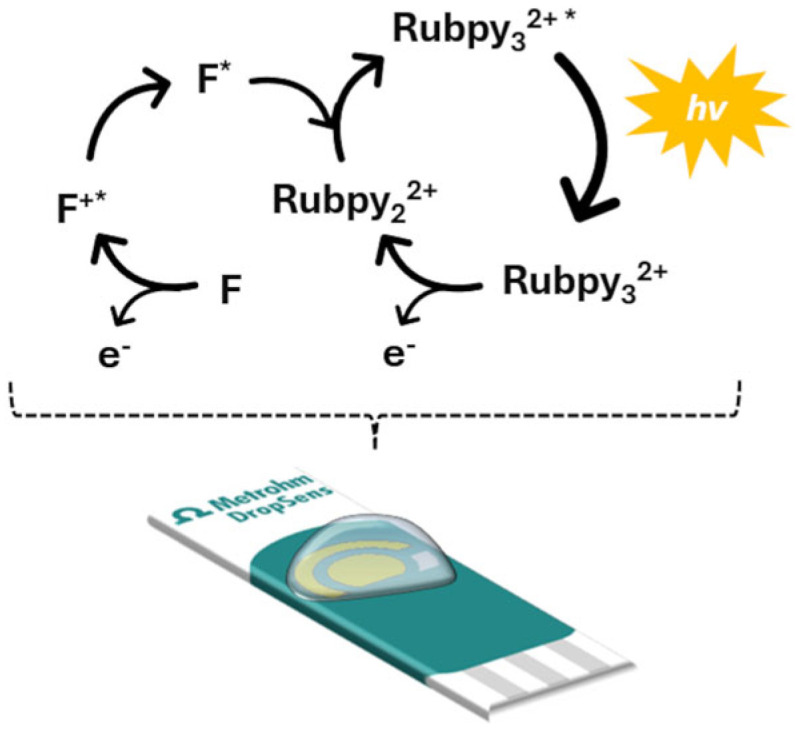
ECL mechanism with Ru(bpy)_3_^2+^ as luminophore and fentanyl as co-reactant.

Prior to optimization of the experimental conditions, the initial step was to verify the functionality of the proposed ECL system, which uses Ru(bpy)_3_^2+^ as the luminophore and fentanyl as the co-reactant, as is shown in Figure 2. This preliminary validation was essential to confirm that the electrochemical excitation of the luminophore in the presence of fentanyl leads to detectable light emission, thereby demonstrating the usefulness of the proposed system for analytical purposes.

**Figure 3:**
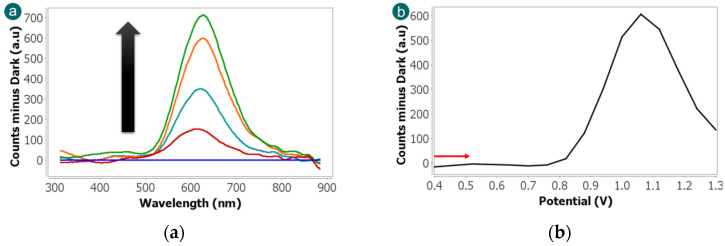
(**a**) ECL spectra and (**b**) evolution of 620 nm band with potential obtained scanning the potential from +0.40 V to +1.30 V at 0.05 V·s^−1^ in 2.5 × 10^−3^ M Ru(bpy)_3_^2+^ and 1 × 10^−4^ M fentanyl in 0.1 M PBS solution. Emission spectra were collected with a microspectrometer detector, using an integration time of 3 s.

Initially, the ECL measurement was carried out using the microspectrometer cell, since this detector allow collecting visible spectra simultaneously with the electrochemical reaction. This device allows the direct observation of the spectral features associated with the luminophore, allowing the differentiation of the characteristic band of Ru(bpy)_3_^2+^ centred around 620 nm [37]. The experiment was performed scanning the potential from +0.40 V to +1.30 V at 0.05 V·s^−1^ in a solution containing 2.5 × 10^−3^ M Ru(bpy)_3_^2+^ and 1 × 10^−4^ M fentanyl in 0.1 M PBS (pH 6). As shown in Figure 3a, a well-defined emission band is clearly detected at 620 nm. A blank experiment was performed without fentanyl and no ECL bands were detected. This control experiment allows us to conclude that fentanyl acts as a suitable co-reactant with Ru(bpy)_3_^2+^ as the luminophore since the luminescence signal is exclusively associated with the ruthenium complex. This specificity is advantageous for analytical applications since it reduces spectral interference and simplifies signal interpretation. The absence of secondary bands also implies that fentanyl does not contribute directly to the emission but rather facilitates the redox reactions necessary for luminophore excitation.

As can be observed in Figure 3b, further insight into the ECL mechanism was obtained by analysing the evolution of the 620 nm emission band with the applied potential. At lower potentials (from +0.40 V to +0.76 V), no luminescence bands were detected, indicating that the oxidation of Ru(bpy)_3_^2+^ is not produced. However, when the potential increases, the ECL signal at 620 nm is observed and it reaches the maximum intensity at +1.06 V. This potential corresponds to the electrochemical oxidation peak of the Ru(bpy)_3_^2+^ and it confirms that the generation of the excited state and thus the light emission are closely linked to the redox behaviour of the luminophore.

These findings validate the fundamental operation of the ECL system and establish a reliable baseline for subsequent optimization. The ability of fentanyl to act as a co-reactant in this context opens the door to its detection via ECL due to the strong and stable luminescence of Ru(bpy)_3_^2+^. Moreover, the clear correlation between applied potential and emission intensity provides a robust framework for tuning the system’s sensitivity and dynamic range in future experiments.

### 3.2. Optimization of Experimental Parameters

Once the performance of the ECL emission was verified under standard conditions, a systematic study was carried out to enhance the intensity and reliability of the luminescent signal. This process allows us to identify the most favourable experimental parameters to maximize the ECL response, thereby improving the sensitivity of the detection method. The optimization process was conducted using a photodiode-based detection system. This detector was selected for its high sensitivity, rapid response time, and suitability for quantitative ECL measurements.

#### 3.2.1. pH Buffer Solution

**Figure 4:**
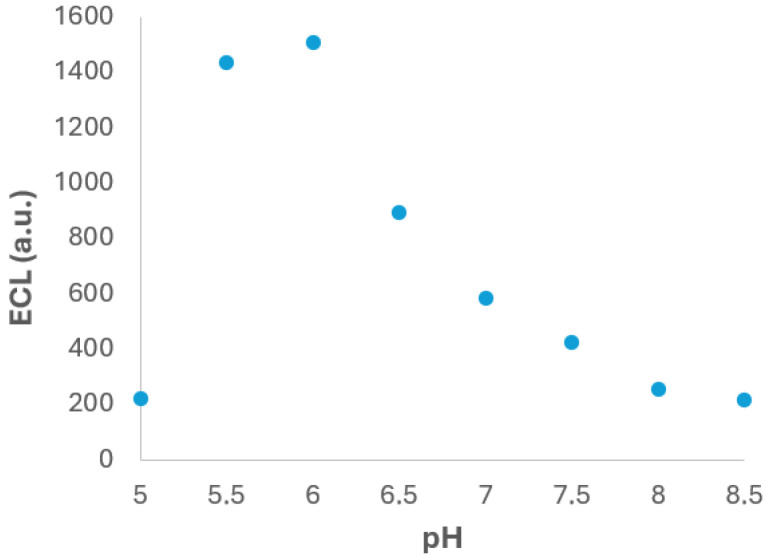
ECL signal vs. the pH PBS solution. Experiments were performed in 2 × 10^−4^ M Ru(bpy)_3_^2+^ and 1 × 10^−4^ M fentanyl in 0.1 M PBS solution. Potential was scanned from +0.40 V to +1.30 V at 0.05 V·s^−1^. ECL response was obtained with the photodiode detector.

One of the most critical parameters that influences the ECL measurement is the pH of the supporting electrolyte. The pH can significantly affect the electrochemical behaviour of both the luminophore and the co-reactant, as well as the stability and reactivity of the intermediates involved in the light-emitting reaction. In order to investigate this effect, the test experiments were carried out using PBS solutions with pH values from 5.0 to 8.5 and containing 2 × 10^−4^ M Ru(bpy)_3_^2+^ and 1 × 10^−4^ M fentanyl. The experimental results, summarized in Figure 4, reveal a clear dependence of the ECL signal on the pH of the medium. Specifically, the ECL intensity increases with pH from 5.0 to 6.0, reaching a maximum at pH 6.0. Beyond this point, a gradual decrease in ECL signal was observed as the pH increased up to 8.5. The observed behaviour can be attributed to the acid–base properties of fentanyl, which contains amino functional groups that undergo protonation–deprotonation equilibria depending on the pH. At pH 6.0, fentanyl exists in a favourable balance between its protonated and neutral forms, which could enhance its electrochemical oxidation and facilitates the generation of reactive intermediates necessary for ECL mechanism [38]. At lower pH values, excessive protonation could hinder the electron transfer reaction, while at higher pH values, the reduced availability of protonated species may limit the generation of key intermediate.

Based on these findings, pH 6.0 was selected as the optimal value for subsequent experiments, as it provides the highest ECL response under the tested conditions. This adjustment is expected to enhance the sensitivity and reliability of the ECL-based detection method for fentanyl.

#### 3.2.2. Ru(bpy)_3_^2+^ Concentration

In addition to the pH, the influence of the luminophore concentration on the ECL response was analysed to determine the optimal conditions. This parameter is crucial since the concentration of the luminophore directly affects the efficiency of the ECL reaction and, consequently, the sensitivity of the detection system. To evaluate this effect, a systematic study was performed to evaluate the effect of Ru(bpy)_3_^2+^ concentration on the ECL response. To assess this parameter, a series of experiments were performed in PBS buffer at pH 6.0, previously identified as the optimal pH for ECL emission, with a fixed concentration of 1 × 10^−4^ M fentanyl. The concentration of Ru(bpy)_3_^2+^ was varied from 1 × 10^−4^ M to 1 × 10^−2^ M, and the ECL intensity was recorded using the photodiode detection system under identical electrochemical conditions.

**Figure 5:**
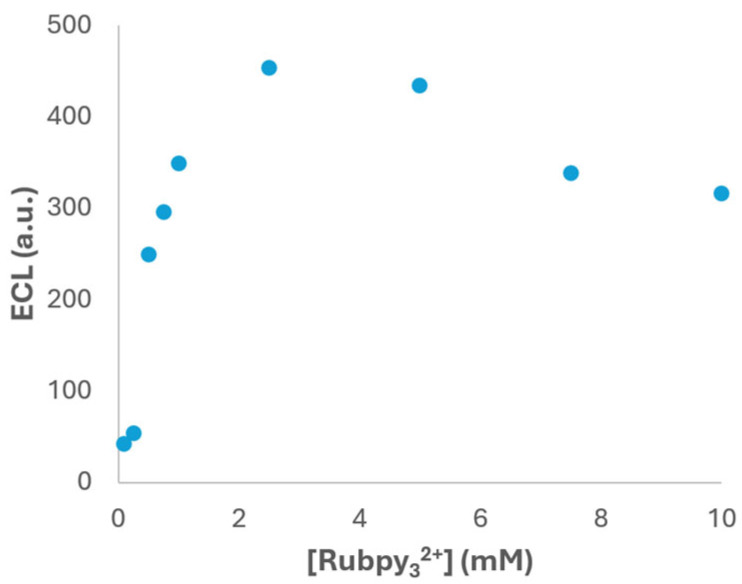
ECL signal vs. Ru(bpy)_3_^2+^ concentration in 1 × 10^−4^ M fentanyl and PBS (pH 6) solution. Potential was scanned from +0.40 V to +1.30 V at 0.05 V·s^−1^. ECL response was obtained with the photodiode detector.

As shown in Figure 5, the ECL signal exhibits a clear dependence on the luminophore concentration. Initially, the intensity increases with the Ru(bpy)_3_^2+^ concentration, reaching a maximum at 2.5 × 10^−3^ M. This enhancement can be attributed to the greater availability of luminophore molecules participating in the redox processes that lead to light emission. However, beyond this concentration value, a decrease in ECL intensity was observed. This behaviour is likely due to self-quenching phenomena, where excessive luminophore molecules in proximity can deactivate excited states through non-radiative pathways. Additionally, inner filter effects may occur at higher concentrations, where the emitted light is reabsorbed by surrounding luminophore molecules before reaching the detector, leading to signal attenuation [39]. Based on these results, the selection of 2.5 × 10^−3^ M Ru(bpy)_3_^2+^ as the optimal concentration provides the highest ECL signal under the tested conditions and represents a compromise between maximizing the efficiency and minimizing adverse effects.

Therefore, by fine-tuning the luminophore concentration, the system can achieve stronger and more consistent signals, which are essential for accurate quantification of fentanyl in complex samples. Moreover, these findings provide valuable insights into the design of future ECL assays, particularly those involving other luminophores or co-reactants with similar electrochemical behaviour.

#### 3.2.3. Working Electrode

Furthermore, considering that the ECL signal is highly dependent on the nature of the working electrode, a comparative study was conducted to evaluate the performance of different electrode materials. The choice of electrode material can significantly influence the electrochemical behaviour of the luminophore and co-reactant, as well as the efficiency of excited-state formation and light emission. Factors such as surface roughness, porosity, conductivity, and chemical composition all contribute to the ECL response.

**Figure 6:**
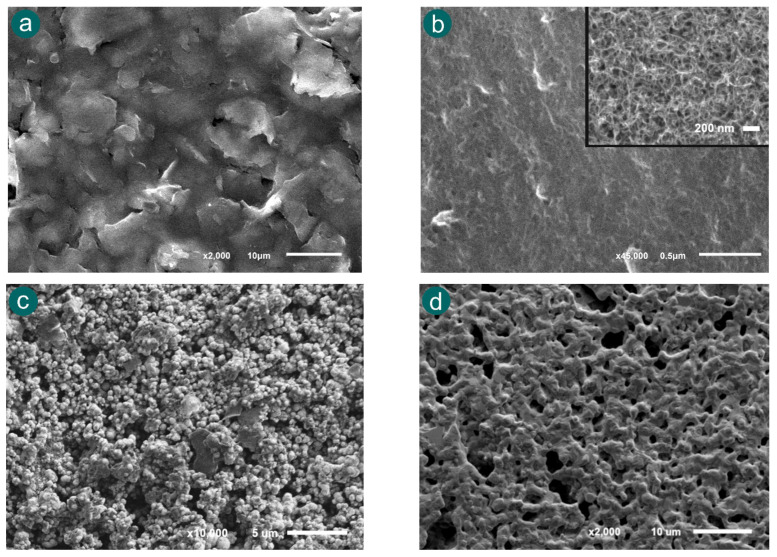
SEM image of (**a**) carbon, (**b**) SWCNTs, (**c**) platinum and (**d**) gold SPEs, respectively.

In this way, four types of SPEs were selected for the evaluation of their morphology: carbon (110), single-walled carbon nanotubes (110SWCNTs), platinum (550BT), and gold (220AT) electrodes were characterized by scanning electron microscopy (SEM). Figure 6 shows the different roughness and porosities of the SPEs selected. As is shown in Figure 6a, the SEM image of 110 SPE displays a relatively smooth surface with moderate roughness and limited porosity. This morphology typically provides a stable platform for electrochemical reactions, which can favour the ECL measurements. Figure 6b shows the morphology of 110SWCNT SPE, which exhibits a highly porous and fibrous structure. The inset image at higher magnification highlights the dense network of nanotubes, offering a significant increase in the surface area. According to these properties, this electrode was considered since the higher area could improve the electrochemical conditions, and it may potentially produce stronger ECL signals. Regarding the platinum 550BT electrode, the SEM image (Figure 6c) shows a granular and rough surface texture. Platinum is known for its excellent catalytic properties and high conductivity, which can facilitate efficient redox reactions. The observed surface features may contribute to localized enhancement of the ECL response due to increased electroactive area. Finally, gold 220AT SPE was characterized in Figure 6d. The image displays a relatively smooth and compact morphology with moderate roughness. In addition, gold electrodes are valued for their chemical stability. These properties provide a good balance for the enhancement of the ECL signal.

These morphological differences suggest that the electrode material and surface structure play a crucial role in modulating the ECL signal. Electrodes with higher roughness and porosity, such as SWCNTs and platinum, are likely to provide more active sites for electron transfer and intermediate formation, thereby enhancing the luminescent emission. On the other hand, smoother electrodes such as carbon and gold surfaces may offer lower background noise and better reproducibility. This comparative analysis between the surface and the ECL signal is essential for identifying the most suitable electrode material for fentanyl detection using the proposed ECL method.

**Figure 7:**
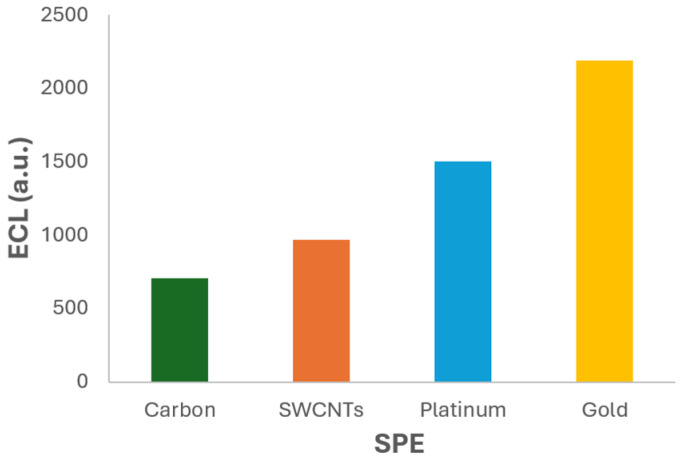
ECL signal vs. SPEs. Experiments were performed in 2.5 × 10^−3^ M Ru(bpy)_3_^2+^ and 5 × 10^−6^ M fentanyl in 0.1 M PBS (pH 6) solution. ECL response was obtained with the photodiode detector.

In this way, the SPEs were tested in order to analyse their influence on the ECL process. All ECL measurements were performed under identical experimental conditions to ensure comparability. The test solution consisted of 2.5 × 10^−3^ M Ru(bpy)_3_^2+^ and 2 × 10^−6^ M fentanyl in 0.1 M PBS at pH 6.0, which had previously been identified as the optimal conditions. The same linear sweep voltammetry protocol was applied to each electrode, and the resulting ECL intensity was recorded using the photodiode detection system. The comparative results are presented in Figure 7 and Figure S1, which illustrate the ECL signal generated by each electrode. A clear variation in luminescent intensity is observed across the different materials, highlighting the critical role of electrode composition and surface properties in the ECL response.

The results clearly demonstrate that the gold SPE (220AT) produces the highest ECL signal among the tested materials. Quantitatively, the ECL intensity obtained with the gold electrode was approximately 3 times higher than that of the carbon electrode, 2.3 times higher than the SWCNTs electrode, and 1.5 times higher than the platinum one. These findings underscore the superior performance of the gold SPE in facilitating the ECL reaction under the tested conditions.

The enhanced ECL response observed with the gold electrode can be attributed to several favourable surface properties. In addition to its excellent electrical conductivity and chemical stability, gold exhibits high reflectivity, which may contribute to more efficient light collection and emission during the ECL process [40]. The smooth and compact morphology of the gold surface, as observed in the SEM analysis (Figure 6d), may also promote electron transfer and reduce signal variability. Moreover, the inertness and compatibility of this material with biological environments make it an attractive choice for applications involving clinical or forensic samples. Its ability to provide strong ECL signals without requiring surface modification simplifies the experimental protocol and enhances the usefulness of the method for routine analysis. According to these results, the gold SPE (220AT) was selected as the optimal working electrode for subsequent experiments. Its better performance in terms of emission intensity makes it the most sensitive platform for ECL-based detection of fentanyl under the tested conditions. This selection is expected to improve the analytical capabilities of the method, enabling more accurate and reliable quantification of fentanyl.

### 3.3. Fentanyl Detection

**Figure 8:**
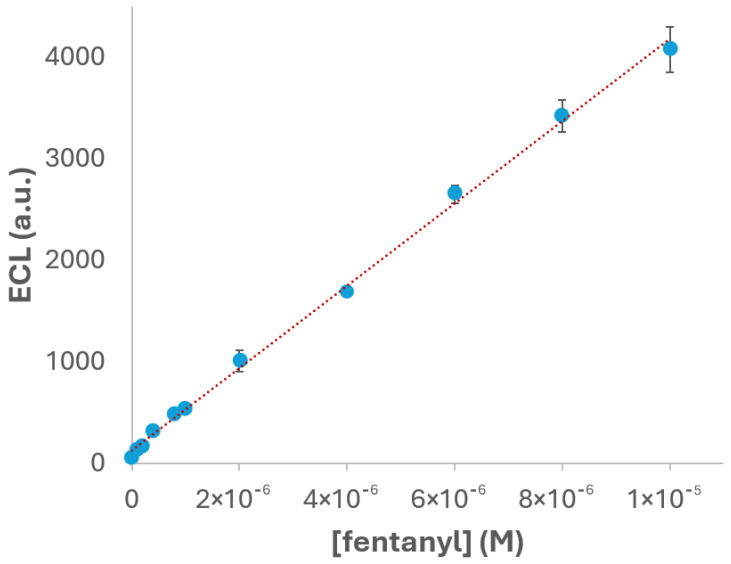
Calibration curve for fentanyl in 2.5 × 10^−3^ M Ru(bpy)_3_^2+^ and 0.1 M PBS (pH 6) solution. The potential was scanned from +0.40 V to +1.30 V, at 0.05 V·s^−1^ using gold SPEs. ECL response was obtained with the photodiode detector.

Under the experimental conditions previously optimized, PBS pH 6, 2.5 × 10^−3^ M Ru(bpy)_3_^2+^ as the luminophore concentration and gold SPEs as the working electrodes, the detection of fentanyl was carried out to evaluate the analytical performance of the proposed ECL system. As displayed in Figure 8, the ECL intensity clearly increases with the evaluated drug concentration, and it exhibits a linear relationship with the concentration of fentanyl within the range of 1 × 10^−7^ to 1 × 10^−5^ M. This linear behaviour is a key indicator of the reliability and suitability of the proposed method, being essential for analytical applications. The experimental data fit the calibration equation y = 4.05 × 10^8^x + 128.01, where y represents the ECL intensity and x the fentanyl concentration. The high correlation coefficient (R^2^ = 0.998) confirms the excellent linearity of the system, indicating that the ECL response is highly predictable and consistent across the tested concentration range. The reproducibility was also evaluated by performing triplicate measurements (n = 3) at each concentration. The relative standard deviation (RSD) was calculated to be 3.7%, demonstrating good repeatability, low variability between replicates and, in this way, the robustness of the proposed method.

To further evaluate the sensitivity of the system, the limit of detection (LOD) was calculated using the standard approach based on three times the standard deviation of the blank signal (3σ) divided by the slope of the calibration curve. The resulting LOD value was 6.7 × 10^−8^ M, highlighting the sensitivity of the method for fentanyl detection under the optimized conditions. This low detection limit, combined with the excellent linearity and reproducibility, suggests the proposed ECL system as a promising tool for the easy, fast and sensitive quantification of fentanyl.

Table 1 summarizes the figure of merits of the electrochemical and ECL-based sensors previously reported for fentanyl detection. As observed, all developed sensors require one or more modifications of the working electrode surface or involve additional treatment steps. In this way, the implementation of commercial SPEs presents an excellent advantage in the development of new sensors. Unlike sensors that rely on laborious surface modification protocols and the incorporation of lab-synthesized functional materials, commercial SPEs offer a ready-to-use platform that significantly reduces fabrication complexity and variability and offers rapid and cost-effective devices.

To evaluate the selectivity of the developed ECL system towards fentanyl detection, a series of potential interfering substances commonly present in biological fluids (ascorbic acid, caffeine, glucose, and urea) were tested. Additionally, acetaminophen, a frequent adulterant found in street drug formulations, was included in the study. ECL measurements were carried out according to the electrochemical protocol previously described and using a solution containing 2.5 × 10^−3^ M Ru(bpy)_3_^2+^, 1 × 10^−6^ M fentanyl, 1 × 10^−5^ M of the interfering compound and 0.1 M PBS (pH 6.0). The presence of these interferents does not produce any significant variation in the ECL response. In all cases, the deviation of the emission signal remains below 5% with respect to the blank experiment, and this demonstrates that the ECL system shows high selectivity for fentanyl under the tested conditions. These results highlight the specificity of the optimized ECL method, making it suitable for future applications.

## 4. Conclusions

In this study, an ECL-based method was successfully developed and optimized for the sensitive detection of fentanyl. The proposed system consists of Ru(bpy)_3_^2+^ as the luminophore and fentanyl as the co-reactant, with gold screen-printed electrodes (220AT) as the working electrodes. The systematic evaluation of the experimental parameters such as PBS buffer pH, luminophore concentration, and electrode material allows the identification PBS pH 6.0, 2.5 × 10^−3^ M Ru(bpy)_3_^2+^ and gold SPEs as the optimal conditions for the system under study.

The initial verification experiments confirmed that fentanyl acts as an effective co-reactant, enabling strong and selective ECL emission of Ru(bpy)_3_^2+^. Optimization of the buffer pH revealed that pH 6.0 provides the most favourable environment for intermediate formation and luminophore excitation. Similarly, the luminophore concentration study demonstrated that 2.5 × 10^−3^ M Ru(bpy)_3_^2+^ yields the highest ECL intensity and avoids quenching effects observed at higher concentrations. The analysis of electrode materials showed that the gold SPE produces the highest ECL signal in comparison with carbon, SWCNT and platinum electrodes. This better performance was attributed to the favourable surface properties of gold, including its reflectivity and electrochemical stability, which enhance light emission and signal reproducibility. Under optimized conditions, the ECL intensity exhibited a linear relationship with fentanyl concentration in the range of 1 × 10^−7^ to 1 × 10^−5^ M, with a correlation coefficient of R^2^ = 0.998 and a relative standard deviation of 3.7%. The calculated limit of detection (LOD) was 6.7 × 10^−8^ M, demonstrating the high sensitivity of the method.

Hence, the proposed ECL system offers a rapid, sensitive, selective and reproducible approach for fentanyl detection, with potential applications in clinical diagnostics, forensic analysis, and harm reduction strategies. Its simplicity (bare commercial SPE from Metrohm DropSens, Spain, without any surface modification) and compatibility with portable platforms make it a promising alternative to conventional detection methods, particularly in scenarios requiring real-time monitoring and trace-level quantification.

## Figures and Tables

**Table 1:** Comparison of the performance of the proposed ECL system based on bare SPEs with the literature on fentanyl electrochemical detection.

Sensor	Technique	LOD (µM)	Linear Range (µM)	Ref.
Glass/SWCNT	DPV	0.011	0.01–1	[23]
Carbon SPE/Zn(II)-MOF	DPV	0.3	1–100	[24]
Carbon SPE/Carbon nano-onions	DPV	0.3	1–60	[25]
Carbon SPE/MWCNT	DPV	0.02	0.064–3.62	[26]
Carbon SPE/MWCNT and ionic liquid	SWV	10	10–100	[27]
Carbon SPE/Ionic liquid	CSWV	5	10–100	[28]
Microneedle/Carbon paste	CSWV	0.025	20–200	[29]
Graphite/MWCNT and NiO nanodisks	DPV	0.0067	0.01–800	[30]
Laser carbonized electrode	SWV	1	20–200	[31]
GCE/MWCNT	DPAdSV	0.1	0.5–100	[32]
Carbon SPE/fCNF	DPV	0.075	0.125–10	[33]
GCE/ MWCNT-HA/Cu-H3BTC	DPV	0.03	0.01–100	[34]
GCE/Ionic liquid	ECL	0.085	0.01–100	[36]
Gold SPE	ECL	0.067	0.1–10	This work

MOF = metal–organic framework; GCE = glassy carbon electrode; fCNFs = functionalized carbon nanofibers; MWCNT-HA = multi-walled carbon nanotube, hydroxyapatite and copper-based MOF; DPV = Differential Pulse Voltammetry; SWV = Square Wave Voltammetry; CSWV = Cyclic SWV; DPAdSV = Differential Pulse Adsorptive Stripping Voltammetry.

## Data Availability

The data presented in this study are available on request from the corresponding author. The data are not publicly available due to privacy.

## Supplementary Materials

The following supporting information can be downloaded at https://www.mdpi.com/article/10.3390/bios15100697/s1, Figure S1: ECL signal obtained using different SPEs.
