# Supplementary material for: Direct ECL Detection of Fentanyl Drug with Bare Screen-Printed Electrodes
Source: Biosensors (Basel). 2025 Oct 15;15(10):697. doi: 10.3390/bios15100697 (PMC12563633; doi:10.3390/bios15100697)
Supplement: Supplementary file 1 [file biosensors-15-00697-s001.zip › biosensors-3903571-supplementary.pdf]

biosensors

Supporting Information

Direct ECL Detection of Fentanyl Drug with Bare Screen-Printed Electrodes

David Ibáñez *, María Begoña González-García, David Hernández-Santos, and ablo Fanjul-Bolado *

Metrohm DropSens S.L.U., Parque Tecnológico de Asturias, C/Faya 28, 33428 Llanera (Asturias), Spain.
*Correspondence: david.ibanez@metrohm.com; pablo.fanjul@metrohm.com.

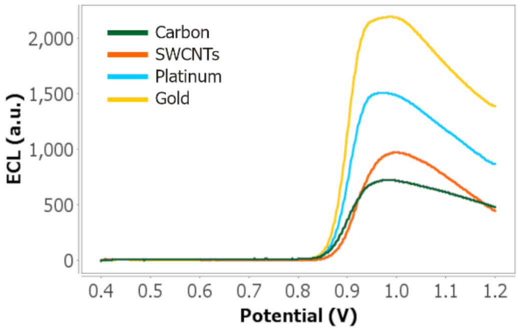

Figure S1. ECL signal obtained using different SPEs: carbon (green line), SWCNTs (orange line), platinum (blue line) and gold (yellow line). Experiments were performed in 2×10^{-3} M $Ru(bpy)_3^{2+}$ and 5×10^{-6} M fentanyl in 0.1 M PBS (pH 6) solution. ECL response was obtained with the photodiode detector.
